# Complementary and Alternative Medicine for the Management of Cervical Radiculopathy: An Overview of Systematic Reviews

**DOI:** 10.1155/2015/793649

**Published:** 2015-08-05

**Authors:** Xu Wei, Shangquan Wang, Jinxue Li, Jinghua Gao, Jie Yu, Minshan Feng, Liguo Zhu

**Affiliations:** ^1^Department of Scientific Research, Wangjing Hospital, China Academy of Chinese Medical Sciences, Huajiadi Street, Chaoyang District, Beijing 100102, China; ^2^Department of General Orthopedics, Wangjing Hospital, China Academy of Chinese Medical Sciences, Huajiadi Street, Chaoyang District, Beijing 100102, China; ^3^Department of Spine, Wangjing Hospital, China Academy of Chinese Medical Sciences, Huajiadi Street, Chaoyang District, Beijing 100102, China

## Abstract

*Background*. Complementary and alternative medicine (CAM) is widely applied in the clinical practice of neck pain owing to cervical radiculopathy (CR). While many systematic reviews exist in CAM to improve CR, research is distributed across population, intervention, comparison, and setting. *Objective*. This overview aims to summarize the characteristics and evaluate critically the evidence from systematic reviews. *Methods*. A comprehensive literature search was performed in the six databases without language restrictions on February 24, 2015. We had identified relevant systematic reviews that examined the subjects with neck pain due to cervical radiculopathy undergoing CAM. Two authors independently appraised the methodological quality using the revised assessment of multiple systematic reviews instrument. *Results*. We had included eight systematic reviews. The effectiveness and safety of acupotomy, acupuncture, Jingfukang granule, manual therapies, and cervical spine manipulation were investigated. Based on available evidence, the systematic reviews supported various forms of CAM for CR. Nevertheless, the methodological quality for most of systematic reviews was low or moderate. In addition, adverse reactions of primary studies were infrequent. *Conclusions*. Current systematic reviews showed potential advantages to CAM for CR. Due to the frequently poor methodological quality of primary studies, the conclusions should be treated with caution for clinical practice.

## 1. Introduction

Cervical radiculopathy (CR) was defined as neck pain in a radicular patter in one or both upper extremities related to compression and/or irritation of one or more cervical nerve roots. A retrospective epidemiology study presented that the annual incidence rate of CR was 83.2 per 100,000 populations. It was reported that up to 80% of CR patients always complained of neck pain, and it would be more and more serious over time [1–3]. For those patients with recurrent condition after initial onset, pain became increasingly frequent. Also, neck pain was a common presenting symptom, which often caused limited cervical range of motion and poor quality of life.

The majority of patients chose conservative, nonoperative treatment course. The main objects of conservative treatments were to relieve pain, improve function, and enhance quality of life [4, 5]. However, a latest systematic review showed that conservative therapies including physiotherapy, collar, and traction were not superior to other interventions on the basis of low-level evidence [6]. Many patients whose symptoms were refractory to conservative treatments and had to undergo surgical therapy probably continued to suffer from neck pain. As an adjunct therapy, complementary and alternative medicine (CAM) approach might help patients improve neck discomfort resulting from CR. At present, the patients usually turn to CAM, which might be considered in rational and individual approach based on the first general rule in medicine “not to harm,” and mainly to treat pain [7, 8]. Meanwhile, many clinicians are reluctant to use these conventional drugs and instead to use CAM approaches, including massage, manipulation, mobilization, exercise, herbal medicines, acupuncture, and cognitive behavioral approach, which are increasingly favored by patients with the hope of alleviating pain-related symptoms with few adverse events [9–14]. However, CAM approaches are not totally without side effects. For instance, patients who receive manipulation treatment or take Chinese herbal medicines may experience dizziness, nausea, vomiting, and other serious risks [15, 16]. Therefore, potential relative benefits or harms of different CAM interventions for CR are worth considering.

Systematic reviews have become a standard method in assessing and summarizing primary studies. With the increasing published systematic reviews of CAM for CR, some interventions are appealing and have been tested in clinical trials. While many systematic reviews exist on CAM to improve CR, research is distributed across population, intervention, comparison, and setting. Therefore, the methodological quality of the reviews is variable and should routinely be appraised. It is necessary to summarize the characteristics and evaluate critically the evidence from systematic reviews in order to give optimal suggestions to future research and clinical practice. The purpose of this overview is to evaluate critically the methodological quality of systematic reviews regarding using CAM to treat CR. To our knowledge, this is the first one which systematically reviewed available systematic reviews of CAM on neck pain due to CR.

## 2. Methods

### 2.1. Inclusion Criteria and Exclusion Criteria

All the systematic reviews or meta-analyses had to pertain to the effective or safety of one or multiple CAM modalities, to focus on CR and include evidence from at least one controlled clinical trial. Patients were diagnosed with cervical radiculopathy, regardless of duration of illness. The interventions were CAM or CAM in combination of existing conventional therapies. According to the World Health Organization definition, CAM was described as a comprehensive term used to refer to both traditional medical systems such as traditional Chinese medicine, Indian Ayurveda, Arabic Unani medicine and various forms of indigenous medicine [17]. Various CAM interventions related in the Cochrane library included alternative medical systems (e.g., Chinese herbal drugs, homeopathy), natural product based therapies (e.g., diet therapy, dietary supplements), energy therapies (e.g., acupuncture therapy, electric stimulation therapy), manipulative and body-based methods (e.g., Chiropractic manipulation, massage), and mind-body interventions (e.g., hypnosis, sensory art therapies, Tai Chi, and Yoga) [18]. Among all the CAM treatments, acupuncture, manual therapy, spinal manipulation, mobilization, and soft tissue massage were the most common CAM treatments in the management of neck pain [19, 20]. Systematic reviews with any pain-related outcome measures were included. Narrative reviews, editorials, commentaries, and letters to the editor were excluded.

### 2.2. Database and Search Strategies

Electronic literature searches were conducted to identify systematic reviews of CAM for CR. The following six electronic databases were searched from their inception through February 24, 2015: PubMed, EMBASE, the Cochrane Central Registry of Controlled Trials (CENTRAL), Chinese Biomedicine (CBM), Allied and Alternative Medicine Database (AMED), and Cumulative Index to Nursing and Allied Health Literature (CINAHL).

The full search strategy of PubMed was presented as follows:

#1 Search ((((cervical radiculopathy [Title/Abstract]) OR cervicobrachial neuralgia [Title/Abstract]) OR cervicobrachial pain [Title/Abstract]) OR neck pain [Title/Abstract]),
#2 Search (((((((((((((((((((((((((((((((((((((((Alternative[Title/Abstract]) OR Acupuncture[Title/Abstract]) OR Alexander technique[Title/Abstract]) OR Aromatherapy[Title/Abstract]) OR Arts therapy [Title/Abstract]) OR Ayurveda[Title/Abstract]) OR traditional Chinese medicine[Title/Abstract]) OR Chiropractic[Title/Abstract]) OR Complementary[Title/Abstract]) OR Dietary supplements[Title/Abstract]) OR Diet therapy[Title/Abstract]) OR Electric stimulation[Title/Abstract]) OR Energy therap^*^[Title/Abstract]) OR Exercise[Title/Abstract]) OR Herb[Title/Abstract]) OR Homeopathy[Title/Abstract]) OR Hydrotherapy[Title/Abstract]) OR Kampo[Title/Abstract]) OR Magnetic[Title/Abstract]) OR Manual therapy[Title/Abstract]) OR Manipulati^*^[Title/Abstract]) OR Massage[Title/Abstract]) OR Mind-body intervention[Title/Abstract]) OR Mobilization[Title/Abstract]) OR Non-herbal[Title/Abstract]) OR Physiotherapy[Title/Abstract]) OR phototherapy[Title/Abstract]) OR Reflexology[Title/Abstract]) OR Relax^*^[Title/Abstract]) OR Reiki therapy[Title/Abstract]) OR Qi gong[Title/Abstract]) OR Spiritual healing[Title/Abstract]) OR Shiatsu[Title/Abstract]) OR Tai Chi[Title/Abstract]) OR traditional Korean medicine[Title/Abstract]) OR Therapeutic touch[Title/Abstract]) OR Tui na[Title/Abstract]) OR Ultrasonic therapy[Title/Abstract]) OR Yoga[Title/Abstract]),
#3 Search ((systematic review [Title/Abstract]) OR meta-analysis [Title/Abstract]),
#4 Search (#1 and #2 and #3).

We also contacted content experts and hand-searched a number of journals published in China. No limits were applied for language and foreign papers were translated. Two authors (X. Wei, S. Q. Wang) independently selected the systematic reviews according to the inclusion criteria; disagreements were resolved by discussion and reached consensus through a third party (L. G. Zhu).

### 2.3. Data Extraction and Methodological Quality Assessment

First of all, the extracted information summarized essential characteristics of systematic reviews, including the name of first author, year of publication, sample size of included studies, meta-analysis or not, the intervention and control, clinical outcome, adverse effect, and conclusion for each systematic review.

A measurement tool for the “assessment of multiple systematic reviews” (AMSTAR) was used to evaluate the methodological quality of systematic reviews [21]. The internal and external validity of AMSTAR had been validated. AMSTAR has good agreement, reliability, construct validity, feasibility, and external validity [22, 23]. The tool is also reliable, valid, and easy to use for methodological quality assessment of systematic reviews on Traditional Chinese medicine, as one type of CAM [24]. The eleven items were evaluated: “a priori” design, duplicate study selection and data extraction, comprehensive literature search, the status of publication (i.e., grey literature) used as an inclusion criterion, a list of studies (included and excluded), the characteristics of the included studies, the scientific quality of the included studies, the scientific quality of the included studies used in formulating conclusions, the methods used to combine the findings of studies, the likelihood of publication bias, and the conflict of interests [21]. But AMSTAR failed to produce quantifiable assessments of systematic review quality [17, 25].

On the basis of eleven items of the original instrument, the revised “assessment of multiple systematic reviews” instrument (R-AMSTAR) was developed to quantify the quality of systematic reviews [25]. Each item's score ranges from 1 to 4 (maximum), and the R-AMSTAR total scores has a range of 11 to 44 (maximum). According to the conventional criterion, total score of 22 was considered an acceptable cutoff point [25]. Methodological quality of systematic reviews was classified into three grades in our study: high quality (total score > 33), moderate quality (22 < total  score ≤ 33), and low quality (11 ≤ total  score ≤ 22).

Subsequently, we constructed a data extraction form for this study, in which eleven items of R-AMSATR were adopted directly. Two authors conducted data extraction (J. Yu, M. S. Feng) independently according to the contents. Differences were resolved by discussion and reached consensus through a third reviewer (L. G. Zhu).

## 3. Results

### 3.1. Description of Included Systematic Reviews

**Figure 1:**
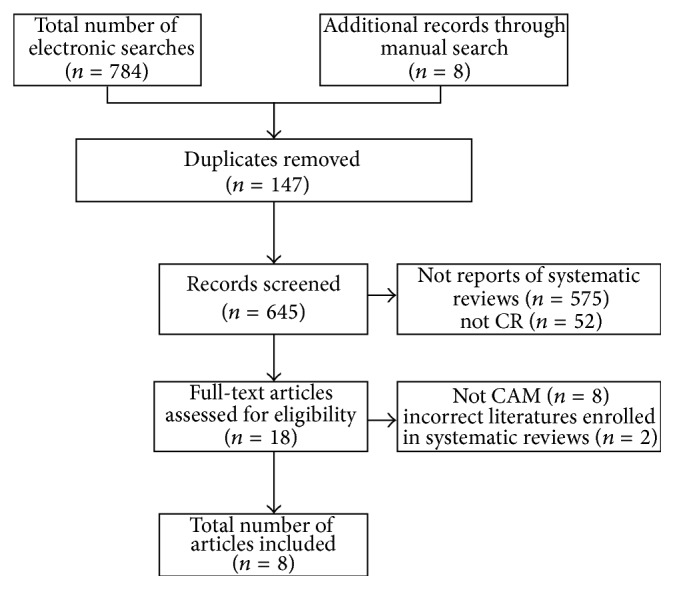
Flow diagram.

Our searches generated 792 articles, of which 784 had to be excluded. The reasons for exclusion were duplicates (*n* = 147), not reports of systematic reviews (*n* = 575), not CR (*n* = 52), and not CAM (*n* = 8). Two articles, which were initially included in the review based on information from the abstracts, were later excluded secondary to incorrect literatures enrolled in systematic reviews and therefore had an insufficient R-AMSTAR score [26, 27]. Thus eight systematic reviews met our eligibility criteria. A flow diagram showed the literature search and screening process (Figure 1). They were published between 2007 and 2015. Seven systematic reviews were published in Chinese [28–34]. One recent systematic review was published in English [35].

### 3.2. Essential Characteristics of Systematic Reviews

The characteristics of the enrolled systematic reviews were summarized in Table 1. One systematic review was about acupotomy [28], other two systematic reviews focused on all kinds of acupuncture (conventional acupuncture, electropuncture, and abdominal acupuncture) [29, 31], and another one was related to a Chinese patent medicine called Jingfukang granule [33]. Additionally, there were three systematic reviews concerning manual therapies, including manipulation, massage, mobilization, and acupressure [30, 32, 34]. The remaining systematic review was about cervical spine manipulation [35].

### 3.3. Methodological Aspects of the Included Reviews

Table 2 provides a formal assessment of the quality of all included systematic reviews. Methodological quality scores of the included reviews ranged from 18 to 36 points according to the R-AMSTAR total scores. Of these systematic reviews, two were of low quality [28, 33], four were of moderate quality [29–32, 34], and one was evaluated high quality [35].

Only one study had “a priori” design [35]. All reviews conducted duplicate study selection and data extraction. Two systematic reviews performed a comprehensive literature search [29, 35]. Almost all the reviews did not use the status of publication as an inclusion criterion and provide a list of included and excluded studies. Items 6–8 (the characteristics of the included studies provided, the scientific quality of the included studies assessed and documented, and the scientific quality of the included studies used appropriately in formulating conclusions) satisfied less than half of the total scores for the majority of systematic reviews. Five systematic reviews used appropriate methods to combine the findings of studies [29–31, 34, 35]. Three systematic reviews assessed the likelihood of publication bias [32–34]. In addition, only one systematic review had statement of sources of support and laid emphasis on whether conflict of interests existed [35].

### 3.4. Acupotomy

Liu et al. aimed to evaluate the quality of clinical study and efficacy of the treatment for CR by acupotomy [28]. Meta-analysis showed that the group of acupotomy was better than that of acupuncture. However, included three studies had some methodological flaws such as inadequate study design, poor reporting of results, and small sample size. In addition, a nonrandomized controlled trial was enrolled in the meta-analysis. Accordingly, there were not enough high grades of evidence recommendation.

### 3.5. Acupuncture

Sun et al. critically assessed the efficacy of acupuncture versus traction in the treatment of CR [29]. The effective rate and improvements in McGill pain questionnaire scores of acupuncture group (including conventional acupuncture, electropuncture, and abdominal acupuncture) were better than traction group, and significant difference was also noted with acupuncture plus traction group versus traction group. But the quality of included studies was partly low.

Hu et al. assessed and compared the clinical effects and safety of acupuncture and traction therapy for CR [31]. Acupuncture (conventional acupuncture or electropuncture) was safe and showed better clinical effect than traction in the treatment of CR. The authors stressed the limitations of the randomized controlled trials included in the analysis and the low methodological quality of the primary studies. The conclusion was not definite due to the low level of evidence eventually.

### 3.6. Jingfukang Granule

Zhang et al. aimed to evaluate the efficacy of Jingfukang granule for patients with CR [33]. The effective rate of Jingfukang group was better than the other groups. Nevertheless, due to a high risk of selection bias and detection bias in the included studies, the evidence was insufficient. Few primary studies prevented firm conclusions.

### 3.7. Manual Therapies

Guo et al. appraised the safety and efficacy of manipulation and massage for treating CR [30]. The results suggested a significant effect of manipulation and massage for the treatment of CR. The authors described that limited primary studies, poor study design, and different treatment methods were the reasons of low quality. In a word, these findings should be treated with caution.

Wang et al. aimed to evaluate the evidence from randomized controlled trials and quasi-randomized controlled trials for the effectiveness of manipulation and massage for CR in detail [32]. The result of meta-analysis showed that both single-application and union-application of manipulation or massage were effective for CR and superior to other treatments. But the heterogeneity of enrolled studies decreased methodological quality and reliability of the conclusion. The authors of the systematic review recommended more rigorous randomized controlled trials.

Yang et al. assessed the efficacy and safety of manual therapies (manipulation, massage, mobilization, and acupressure) for CR [34]. The results of systematic review showed manual therapies had advantages in short-term therapy and performed more efficiently on the long-term treatment, but it was of no statistical significance. In one study, adverse reactions of massage were on record (Table 1). This systematic review reported that the wide variety of therapeutic manual techniques, diagnosis, and treatment standards of CR was inconsistent. The authors were uncertain about the effectiveness of manual therapies and recommended more and better research.

### 3.8. Cervical Spine Manipulation

Zhu et al. evaluated the effectiveness and safety of cervical spine manipulation for CR [35]. Meta-analysis suggested that cervical spine manipulation improving visual analogue scale for pain showed superior immediate effects compared with cervical computer traction. The overall strength of evidence was judged to be moderate quality according to GRADE (grades of recommendation, assessment, development, and evaluation) approach. However, there are still selection bias and attrition bias in the primary studies. Moreover, the adverse event of cervical spine manipulation in treating degenerative CR was not clear.

## 4. Discussion

More recently, CAM has shown high usage in the developed countries, especially for those with chronic diseases, such as neck pain [36–41]. CAM can be the “mainstay” of health care delivery, particularly in remote or rural areas in the developing countries [42]. A multitude of patients suffering from CR used CAM to address their symptoms, including neck pain [8]. Despite significant evidence for the use of CAM on CR into professional clinical practice, it is necessary to use the methods of overview of systematic reviews to summarize available evidence. There was a paucity of reports evaluating the potential for the therapeutic effect and safety of CAM for CR. This paper was aimed at providing an overview of systematic reviews. Eight systematic reviews were included [28–35]. We placed the discussion in the text of existing evidence.

The effectiveness and safety of acupotomy, acupuncture, Jingfukang granule, manual therapies, and cervical spine manipulation were investigated. Based on available evidence, the systematic reviews provided some evidence to support various forms of CAM for CR. All the systematic reviews showed the CAM intervention was superior to the control group, respectively.

In this paper, we used R-AMSTAR to evaluate the quality of systematic reviews. Regrettably, the methodological quality for the majority of the systematic reviews was low or moderate. Those “positive findings” might be unreliable because of the frequently poor quality of previous studies, such as poor study design, small sample size, selection bias, and detection bias. One of the common problems was high heterogeneity across studies, especially in the systematic reviews of acupuncture and manual therapies [29–32, 34]. Wide differing estimates of the treatment effect across individual trials implied true differences in underlying treatment effects [43]. For example, three systematic reviews paid attention to comprehensive effect of manual therapies [30, 32, 34]. But the effect of single manual therapy was not necessarily identical. We suggested that systematic review of single manual therapy which included massage, manipulation, or mobilization for CR should be performed. Another problem was the inappropriate choice of control group in the randomized controlled trials. There was no placebo-controlled study design. Additionally, not all therapies as control group were recommended by the clinical practice guideline [28, 32]. In the future, the randomized controlled trials that compared CAM with placebo or “gold-standard treatment” should be well done. But besides that, primary studies with no randomization, allocation concealment, blinding, outcome not to be measured in a large proportion of patients, patients lost to follow-up, or failure to adhere to the intention-to-treat principle during the analysis were highly susceptible to bias [44–47]. Although the latest systematic review was judged to be high quality, the positive results were presented with limited eligible studies [35]. According to the evidence we collected, we could not recommend any CAM therapeutic option for CR.

Risk assessment was an inherent component of CAM therapy practice as well. Six out of eight systematic reviews mentioned adverse reactions in the overview [29–32, 34]. In the systematic reviews, adverse reactions were infrequent. Two systematic reviews did not report any significant adverse effects or allergic reactions [28, 33]. The total number of adverse reactions after acupuncture was low in two systematic reviews by Sun et al. and Hu et al. [29, 31]. Mild pain and bleeding were observed in a randomized controlled trial [29]. The analysis of the publications indicated that fainting, allergy, and pain were the common adverse reactions. And various causes lead to adverse reactions to acupuncture. So the researchers took the attitude that the safety guidelines for the risk management of acupuncture operation should be established [48, 49]. Meanwhile, as the most commonly used treatment method for CR, published cases of severe adverse events following chiropractic manipulation illustrated the need for the safety evaluation of manual therapies [50, 51]. In this overview we discussed, only one trial reported that neck pain became more serious after massage [30, 34]. Nevertheless, there was no confirmative evidence to identify the safety of other CAM interventions for CR. Further safety testing of CAM therapies, no doubt, was an essential part for future research.

In our opinion, this overview of systematic reviews had some limitations. On the one hand, although comprehensive searches were conducted, there is no guarantee that all relevant systematic reviews were enrolled. Also, we did not include primary randomized controlled trials that evaluated CAM for CR and keep up with the latest research progress. For instance, a new research program about a compound traditional Chinese herbal medicine for neck pain in patients with CR is ongoing [12]. On the other hand, the paucity of primary studies included in systematic reviews influenced conclusions. Only three trials were enrolled in systematic reviews, which was associated with low quality [28, 33, 35]. When studies included relatively few patients and few events occurred, estimates of the effect usually had indeterminate results [43]. To make progress in this area, further high-quality randomized controlled trials are required to prove the role of CAM in the treatment of CR. We also need more effective CAM interventions around the world, better implementation of existing therapies, better quality of reporting, and more reliable systematic reviews.

## 5. Conclusion

In conclusion, current systematic reviews showed potential advantages to CAM for CR in alleviating neck pain or related symptoms. Acupotomy, acupuncture, Jingfukang granule, manual therapies, and cervical spine manipulation were the CAM interventions. The adverse reactions of primary studies were infrequent. However, the safety of other CAM therapeutic methods could not be adequately judged. Our overview suggested that the methodological quality for most of systematic reviews (7/8) was low or moderate. Due to the poor study design of previous studies, small sample size, selection bias and detection bias, and high heterogeneity across studies, these conclusions of available systematic reviews should be treated with caution for future clinical practice.

## Figures and Tables

**Table 1:** Summary of the included studies in the review.

First author (year)	Number of primary studies	Meta-analysis	Intervention	Comparison	Clinical outcome	Adverse effects	Conclusion
Liu, 2007 [28]	3	Yes	Acupotomy	Acupuncture (3 studies)	^*^Cure, markedly effective, effective, ineffective	All trials did not mention whether adverse events have occurred	There are some defects in clinical study on the treatment for CR by acupotomy; treatment for CR by acupotomy is safe and efficient treatment for CR, but the bad quality of articles and the deficiency of methodological decline the efficacy and the reliability

Sun, 2009 [29]	8	Yes	Electropuncture Acupuncture Abdominal acupuncture Abdominal acupuncture + CCT Acupuncture + CCT + massage Electropuncture + massage + CCT + TDP	CCT (2 studies) CCT CCT CCT CCT + massage (2 studies) massage + CCT + TDP	^*^Cure, markedly effective, effective, ineffective ^*^MPQ	One trial reported the adverse reaction, including mild pain and bleeding	Acupuncture treatment is effective for CR and is superior to traction in the aspects of effective rate and pain alleviating. The curative effect of traction treatment could be improved when combining with acupuncture. However, the conclusion was uncertain because the quality of enrolled papers was partly low

Guo, 2012 [30]	9	Yes	Long's manipulation Manipulation Rotation manipulation Manipulation Manipulation Massage Massage Manipulation	CCT + instruments CCT + IFTA CCT CCT (2 studies) Buluofen tablets CCT CCT + Buluofen CCT + acupuncture	^*^Cure, markedly effective, effective, ineffective	One trial reported the mild adverse reaction	Manipulation or massage treatment on CR is safe, effective and both cure rate and the effective rate are much better than other therapies; but due to the limited number of documents included and the quality being not very high, the conclusion is still uncertain

Hu, 2012 [31]	14	Yes	Acupuncture Electropuncture Acupuncture + CCT Electropuncture + CCT	CCT (3 studies) CCT (3 studies) CCT (4 studies) CCT (4 studies)	^*^Cure, markedly effective, effective, ineffective	Two trials reported the safety, but no adverse reactions were observed	Acupuncture was safe in the treatment of CR. Acupuncture showed better clinical effect than traction therapy. In addition, acupuncture had better analgesic effect and could reduce recurrence. Therefore, acupuncture is probably superior to traction therapy in the treatment of CR, which is not definite due to relatively low level of evidence

Wang, 2013 [32]	28	Yes	Massage Massage + CCT Massage Massage + CCT Manipulation Manipulation Massage Massage Manipulation Massage + TCM capsule Massage + TCM Decoction Manipulation + VCDI Massage Manipulation + acupuncture Manipulation + CCT + TCMI Manipulation + CCT + EH Manipulation + CCT + IFTA	CCT (7 studies) CCT (5 studies) CCT + acupuncture Acupuncture + CCT CCT + TCM Pills CCT + Buluofen Lornoxicam tablets TCM capsule TCM plaster TCM capsule TCM decoction VCDI Acupuncture Acupuncture (2 studies) CCT + TCMI CCT + EH CCT + IFTA	^*^Cure, markedly effective, effective, ineffective ^*^MPQ	Three studies reported the adverse reactions. Only one study described that skin allergy reaction occurred in seven patients after the plaster therapy	Single-application of manipulation or massage was superior to traction group and medicine group in effective rate, while no significant differences were noted between the manipulation or massage group and other control groups. In the trials of comparison between union-application of manipulation or massage and other treatments, only manipulation plus acupuncture versus acupuncture group was not significantly different. However, the conclusion is uncertain because the quality of enrolled papers is partly low

Zhang, 2013 [33]	3	Yes	Jingfukang granule Jingfukang granule Jingfukang granule	CCT + Buluofen Meloxicam tablets TCM granule	^*^Cure, markedly effective, effective, ineffective	No significant adverse effects or allergic reactions were reported	Jingfukang granule was superior to the other therapies. To compare Jingfukang granules with western medicine, there was no significant advantage. So Jingfukang granule was effective in the treatment of CR. However, the evidence is insufficient to determine the effect of Jingfukang granules

Yang, 2013 [34]	30	Yes	Manipulation Massage + acupressure Massage Mobilization Manipulation + acupressure Manipulation + massage	CCT (18 studies) CCT CCT (5 studies) CCT (3 studies) CCT CCT (2 studies)	^*^Cure, markedly effective, effective, ineffective ^*^VAS ^*^TCSSG	Fourteen trials mentioned whether adverse events have occurred. Only one trial showed that ten patients presented red mark left on the cervical skin and the pain getting worse after massage	Manipulation or massage has advantages in treating CR with respect to short-term therapy, pain relief and the signs/symptoms amelioration compared with cervical traction. Manipulation or massage is of higher security. Nevertheless, the wide variety of therapeutic manipulation or massage techniques, diagnosis, and treatment standards is inconsistent

Zhu, 2015 [35]	3	Yes	Cervical spine manipulation	CCT (3 studies)	^*^VAS	One out of three trials reported the adverse events and none with a small sample size	There was moderate level evidence to support the immediate effect of cervical manipulation in treating CR. However, the safety of cervical manipulation cannot be taken as an exact conclusion

^*^Definition of “cure,” “markedly effective,” “effective,” and “ineffective,” cured: clinical symptoms resolved, the cervical or limb functions restored to normal.

Markedly effective: clinical symptoms significantly alleviated, cervical and limb functions effective.

Effective: clinical symptoms alleviated, but cervical or limb functions remain impaired. Ineffective: clinical symptoms and signs remain unchanged after the treatment.

^*^MPQ: McGill pain questionnaire; ^*^VAS: visual analogue scale; ^*^TCSSG: total clinical symptoms and signs grading.

CR: cervical radiculopathy; CCT: cervical computer traction; TDP: special electromagnetic therapeutic apparatus; IFTA: intermediate-frequency therapy apparatus.

TCM: traditional Chinese medicine; VCDI: vertebral canal drug injection; TCMI: traditional Chinese medicine injection; EH: electromagnetic heating.

**Table 2:** Assessment of methodological quality for included systematic reviews.

Study ID	Item 1	Item 2	Item 3	Item 4	Item 5	Item 6	Item 7	Item 8	Item 9	Item 10	Item 11	Total score	Quality
Liu et al., 2007 [28]	BC	ABC	A	0	0	0	0	A	BCE	0	A	18	Low
Sun et al., 2009 [29]	BC	ABC	ABCE	D	0	A	AB	AB	ABCD	0	A	27	Moderate
Guo et al., 2012 [30]	BC	ABC	ABE	0	0	A	AB	AB	ABCE	0	A	25	Moderate
Hu et al., 2012 [31]	C	ABC	AB	0	ABC	A	AB	AB	ABCE	0	A	26	Moderate
Wang et al., 2013 [32]	BC	ABC	AB	0	0	A	AB	AB	A	AB	A	23	Moderate
Zhang et al., 2013 [33]	BC	ABC	AB	0	0	A	A	AB	AB	AB	0	22	Low
Yang et al., 2013 [34]	BC	ABC	ABC	0	0	AB	A	AB	ABCE	AB	A	27	Moderate
Zhu et al., 2015 [35]	ABC	ABC	ABCE	AD	AC	ABC	ABCD	ABC	ABCD	0	AB	36	High

Item 1: was an “a priori” design provided?

Item 2: was there duplicate study selection and data extraction?

Item 3: was a comprehensive literature search performed?

Item 4: was the status of publication (i.e., grey literature) used as an inclusion criterion?

Item 5: was a list of studies (included and excluded) provided?

Item 6: were the characteristics of the included studies provided?

Item 7: was the scientific quality of the included studies assessed and documented?

Item 8: was the scientific quality of the included studies used appropriately in formulating conclusions?

Item 9: were the methods used to combine the findings of studies appropriate?

Item 10: was the likelihood of publication bias assessed?

Item 11: was the conflict of interests stated?

## Abbreviations

CAM: Complementary and alternative medicine
CR: Cervical radiculopathy
AMSTAR: Assessment of multiple systematic reviews
R-AMSTAR: Revised “assessment of multiple systematic reviews” instrument
GRADE: Grades of recommendation, assessment, development, and evaluation
MPQ: McGill pain questionnaire
VAS: Visual analogue scale
TCSSG: Total clinical symptoms and signs grading
CCT: Cervical computer traction
TDP: Special electromagnetic therapeutic apparatus
IFTA: Intermediate-frequency therapy apparatus
VCDI: Vertebral canal drug injection
TCMI: Traditional Chinese medicine injection
EH: Electromagnetic heating.
